# 25, 50 and 75 years ago

**DOI:** 10.1111/ans.18000

**Published:** 2022-09-12

**Authors:** Julian A. Smith

**Affiliations:** ^1^ Department of Surgery Monash University Melbourne Victoria Australia

## 25 years ago

**Gillett D, Kennedy C, Carmalt H. Breast cancer in young women. *ANZ J. Surg.* 1997;67:761–764**

It is believed that cancer of the breast is more difficult to diagnose in young women and it has long been disputed whether breast cancer occurring in women aged ≤40 years is more aggressive than that occurring later in life. A number of reports in the literature suggest that the disease is of similar aggressiveness in the young patients and older age groups, while other reports suggest that it is more aggressive and carries a higher mortality in young women. To address these aspects of breast cancer we have undertaken a review of the cases treated at The Strathfield Breast Centre between 1989 and 1996 and compared the disease in the young and old groups with particular reference to the modes of diagnosis, the pathological staging and types of tumour and the outcomes of treatment. The accuracy of ultrasound and fine needle aspiration biopsy were similar in both groups, but the false negative rate of mammography in the young patients was 15% or 50% greater than that which was observed in the older patients. The incidence of histopathological type, bilaterality, size of lesion and receptor positivity were the same in both groups. In the young group, 40% had Grade 3 tumours compared with 27% in the older group. Nineteen percent of young patients had 4 or more lymph nodes involved while only 10% of the older patients had similar lymph node involvement. Overall 5‐year survival was 79% in the older patients compared with 90% in the young patients. The spectrum of disease is similar in both the young and older patient and the prognosis is no worse for the young group but mammography is less effective in the diagnosis of the young patient.

**Zissiadis Y, Langlands AO, Barraclough B, Boyages J. Breast conservation: long‐term results from Westmead Hospital. *ANZ J. Surg.* 1997;67:313–319**

Breast conservation has been shown to be a safe and effective alternative to mastectomy in early‐stage breast cancer. The present study reviews the long‐term outcome and toxicity after treatment of early breast cancer by conservative surgery and radiation. Between November 1979 and December 1989, 438 patients with Union Internationale Contre le Cancer (UICC) stage I or II breast cancer were treated with conservative surgery and radiation therapy (CS + RT) at Westmead Hospital. Surgery to the breast varied from a local excision to a quadrantectomy, depending on the preference of the referring surgeon. The axilla was surgically dissected in 299 patients (68%). All patients received postoperative breast irradiation. The whole breast was irradiated to 46–54 Gy (median dose, 50 Gy) using 6 Mev photons for 5–6.5 weeks. Boosts were given at the primary tumour site in 336 patients (78%), by electron therapy (88 patients), iridium‐192 (247 patients) or photons (one patient). A total of 44 patients (10%) received adjuvant chemotherapy. The median follow‐up period for surviving patients was 84 months (range: 56–172 months). The 5‐year actuarial rate of local recurrence was 6% (312 patients at risk), and the 10‐year rate was 10% (52 patients at risk). Very young patients (aged 34 years at diagnosis) had a 5‐year actuarial rate of local recurrence of 13% compared to 5% for older patients (*P* = 0.04). Neither the total dose to the primary site nor the boost technique influenced local recurrence. The 5‐year freedom from distant relapse was 83%. The side effects included rib fractures (2%), symptomatic pneumonitis (3%), fatty necrosis or fibrosis requiring surgery (4%), and moderate–severe oedema of the arm (7%). The long‐term data show that CS + RT for UICC stage I or II breast cancer results in low rates of local recurrence which are influenced by age at diagnosis, but not by radiation dose or boost technique. These results confirm those of other international series that CS + RT is a safe alternative to mastectomy for most women with operable breast cancer.

## 50 years ago

**Lewis GM. Oxygen therapy in the surgical patient. *ANZ J. Surg.* 1972;41:280–284**

In recent years attention has been drawn to the effects of surgical and accidental trauma on the microcirculation. Some peripheral vasoconstriction always occurs, and can be seen clinically if it is severe. Pulmonary vasoconstriction also occurs, and reports from several sources suggest that it is much more severe than the systemic response. One thing is certain, and that is that the only way to he sure about any of these patients is to estimate the arterial blood gases. Many people have emphasized the unreliability of clinical signs. Absence of cyanosis does not ensure that oxygenation is adequate. Ross and O'Higgins reported on a man aged 41 years who was not obviously cyanosed even with a Pa0_2_ of 23 mm Hg and a haemoglobin level of 142 g/100 mL. The presence of cyanosis obviously does not depend on the arterial oxygenation. The role of increased cardiac output in preventing it and the possible role of peripheral arteriovenous shunting in producing it, could be subjects for another paper.

**Cullen TH, Popham RR, Voss HJ. Urine cytology and primary carcinoma of the renal pelvis and ureter. *ANZ J. Surg*. 1972;41:230–236**

This is a report of a small series of cases of primary carcinoma of the renal pelvis and ureter, with particular reference to diagnosis of these conditions by cytological examination of the urine. The causes of failure of detection and the advantages of using this method are discussed, and the cytological and histological diagnoses are compared. In summary:

1. False negatives may be expected in patients who have non‐functioning kidneys or obstructed ureters;
2. The persistent finding of degenerate cells indicates partial urinary obstruction in the upper tract or at the bladder neck and neither confirms nor excludes malignancy; and
3. Examination of insufficient satisfactory specimens will increase the false negative rate.

## 75 years ago

**Christie HK. A method for the treatment of fractures of the expanded ends of long bones. *ANZ J. Surg.* 1947;17: 29–36**

**Fig. 1:**
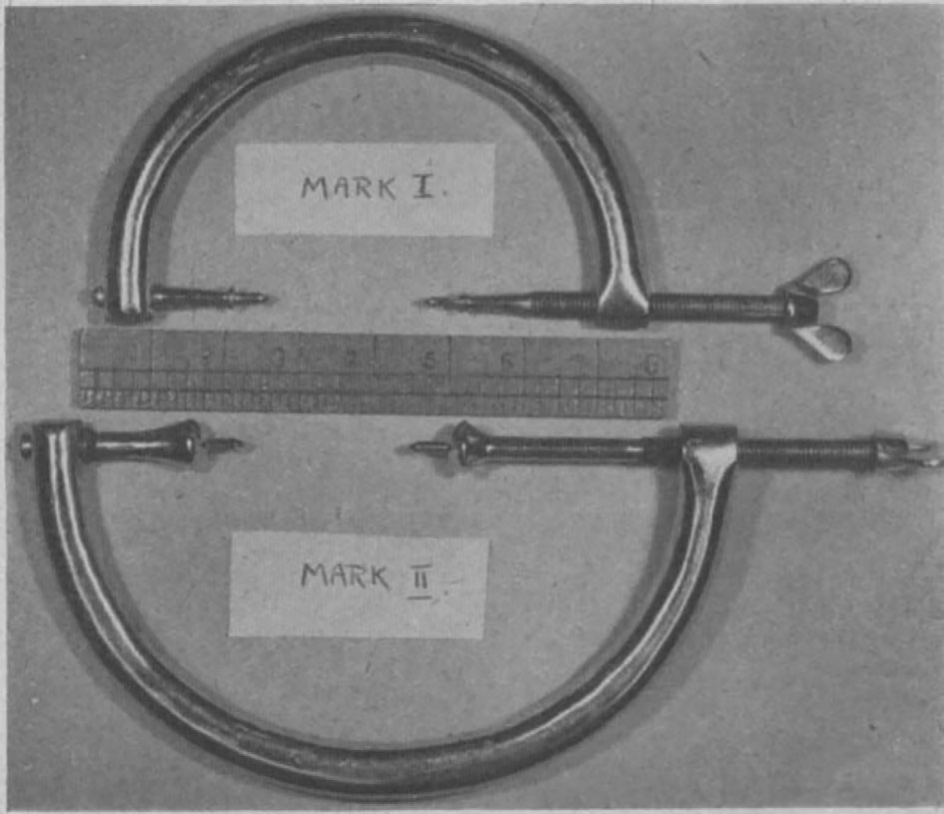
The clamp.

**Fig. 2:**
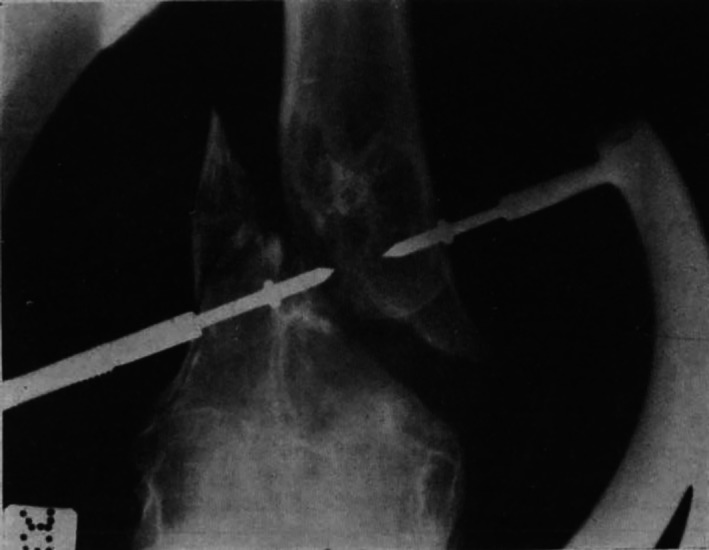
Application of the clamp.

A simple clamping appliance (Fig. 1) for treatment of fractures of the expanded ends of long bones is described. It is a logical expansion of certain well‐known orthopaedic manouvres. The pressure of the clamp is taken by the bone instead of skin, while the points penetrate the bone but do not transfix it. The two‐pointed jaws of the instrument are held rigidly in sockets. One end carries a threaded shank with wings, by the turning of which the clamp is tightened up. The “shoulders” of the jaws are rounded and expand so that they will adapt themselves to any bone contour and will not sink into the cancellous bone. Cases illustrating its use in fractures above and below the knee are described (an example radiograph is shown in Fig. 2). The outstanding advantage of the appliance is close coaptation of the fragments and such firm retention that movements at the joint can be commenced at once. Radioscopic control is required during use of the apparatus.

